# Biotechnological mechanism for improving plant remobilization of phosphorus during leaf senescence

**DOI:** 10.1111/pbi.13212

**Published:** 2019-08-27

**Authors:** Letitia M. Da Ros, Shawn D. Mansfield

**Affiliations:** ^1^ Department of Wood Science University of British Columbia Vancouver BC Canada

**Keywords:** phosphorus, hybrid poplar, *Arabidopsis*, nutrient uptake, nutrient resorption, PHT1 family, senescence, phytoremediation

## Abstract

Phosphorus enrichment of aquatic ecosystems through diffuse source pollution is an ongoing issue worldwide. A potential solution lies in the use of fast‐growing, multipurpose feedstocks, such as trees, to limit the flow of phosphorus into riparian areas through luxury consumption. However, the perennial nature of trees and their use of leaves as storage organs for excess phosphorus may reduce the effectiveness of contaminant removal during periods of leaf abscission. In an attempt to improve phosphorus remobilization during autumnal senescence, transgenic hybrid poplar P39 (*Populus alba* × *Populus grandidentata*) and *Arabidopsis thaliana* harbouring a constitutively expressed low‐affinity potato phosphate transporter (35S::StPht1‐1) were generated using *Agrobacterium*‐mediated transformation. For both species, the highest expressing 35S::StPht1‐1 lines were grown alongside wild‐type plants and subjected to increasing phosphate applications. StPht1‐1 expression in *A. thaliana* led to a reduction in biomass when grown under high‐phosphate conditions and had no effect on phosphate remobilization during senescence. In contrast, StPht1‐1 constitutive expression in P39 resulted in increased leaf phosphate content in the highest expressing transgenic line and minimal to no effect on P resorption efficiency. Surprisingly, sulphate resorption showed the greatest improvement in all three transgenic poplar lines, displaying a 31%–37% increase in resorption efficiency. These results highlight the complexity of nutrient resorption mechanisms in plants.

## Introduction

Due to its lack of mobility between ecosystems, phosphorus is often the most limiting nutrient for growth and is largely responsible for seasonal algal blooms in freshwater systems (Schindler *et al.*, 2008). Phosphorus levels in surface drainage are a product of soil phosphorus, topography, vegetative cover, volume of overland flow and land use (Wetzel, 2001), with inputs from diffuse agricultural sources as the primary cause of reduced water quality (Jarvie *et al.*, 2006; Spiteri *et al.*, 2016). These inputs are difficult to regulate due to the large land base within most agricultural watersheds, affecting the economic and practical feasibility of targeted phosphorus reduction.

Hybrid poplar is a multipurpose feedstock as it has a variety of industrial and ecological uses, including pulp and paper production, oriented strand board fabrication, heating and power generation, a substrate for the production of biofuels, and ecological services in the form of windbreaks and shelterbelts, and phytoremediation. Common traits of hybrid tree species include high‐growth rates and productivity, ease of propagation, and the ability to be coppiced (Isebrands and Richardson, 2014). Coupled with their current use as agricultural shelterbelts, hybrid poplars are an ideal site‐dependent solution capable of safeguarding the multiple entry points available to diffuse source pollutants. Hybrid poplar have demonstrated luxury consumption of phosphorus in excess phosphorus conditions (Da Ros *et al.*, 2018), but have high inorganic phosphate concentrations remaining in leaves after abscission (L.M. Da Ros, R.Y. Soolanayakanahally and S.D. Mansfield, unpublished data). Identification of current barriers to phosphate remobilization could result in more effective phytoremediation of phosphorus‐laden soils.

Nutrient resorption is often studied from an ecological or crop production perspective. Broad‐scale ecological studies of nutrient resorption use cross‐species comparisons to identify which nutrient‐conservation traits are a function of phylogeny and which are predominantly environmental responses (Aerts, 1996; Killingbeck, 1996). In crop science, there is a focus on identifying genes that can increase nutrient‐use efficiency, which includes increased acquisition efficiency and improved remobilization (Han *et al.*, 2015; Veneklaas *et al.*, 2012). Nutrient resorption has been accepted as a widely variable trait that can be affected by an array of determinants, such as age, source–sink relationships, leaching and timing of abscission, depending on the nutrient in question. However, for phosphorus in particular, none of the aforementioned variables have been shown to be correlated with resorption, leaving the controls on phosphorus resorption largely unknown (Chapin and Moilanen, 1991; Killingbeck *et al.*, 1990).

Barriers to phosphorus remobilization are dependent upon where the largest phosphorus storage pools are located *in planta*. Phosphorus in photosynthetic tissues at or above optimal crop concentrations (2–15 mgP/g DW) is stored primarily in the vacuole, followed by nucleic acids, lipids and various ester phosphates (Veneklaas *et al.*, 2012). From experiments completed in barley (*Hordeum vulgare* L.; Mimura *et al.*, 1990), soybeans (*Glycine max* [L.] Merr.; Lauer *et al.*, 1989), sycamore (*Acer pseudoplatanus* L.; Pratt *et al.*, 2009) and *Arabidopsis thaliana* (Pratt *et al.*, 2009), it is apparent that the vacuole stores excess cellular phosphate to buffer cytoplasmic phosphate concentration and sustain cellular metabolism during low phosphate conditions. The perceived mobility of vacuolar phosphate, and the assumption that suboptimal soil phosphate results in low vacuolar phosphate, led much of the historical research to focus on the release of phosphate from the organic phosphorus pool. This involved isolating enzymes required for the hydrolysis for nucleic acids and other phosphate esters, such as RNases and purple acid phosphatases (PAPs). Genes such as AtPAP26 in Arabidopsis were shown to be crucial for efficient phosphorus remobilization and the progression of senescence (Robinson *et al.*, 2012). Once inorganic phosphate is present in the cytosol, the final barrier to transport is the plasma membrane and subsequent loading into the phloem. From previous experiments in poplar (L.M. Da Ros, R.Y. Soolanayakanahally and S.D. Mansfield, unpublished data), inorganic phosphate concentrations remain high in senesced leaves, suggesting a lack of sufficient export from the cell was a primary barrier to resorption. The possibility of increasing phosphate export from the cell via the introduction of exogenous Pht1 transporter was therefore explored.

Members of the Pht1 phosphate transporter family are H^+^/H_2_PO_4_^−^ symporters that span the plasma membrane and show high protein sequence homology both within members and across species (Nussaume *et al.*, 2011). They are considered high‐affinity transporters whose primary function is the uptake of phosphate from the soil matrix; however, studies have shown a range of affinities, revealing their putative roles in phosphate translocation during active growth (Nagarajan *et al.*, 2011; Shin *et al.*, 2004). Of the Pht1 family in *Arabidopsis*, the most plausible candidate to have a role in phosphate remobilization during senescence is AtPht1‐5. AtPht1‐5 has weak expression in roots and high expression in older leaves at the start of senescence (Mudge *et al.*, 2002). Furthermore, 12‐day‐old AtPht1‐5 knockout mutant seedlings show increased shoot and lower root phosphorus content, suggesting reduced remobilization from leaves (Nagarajan *et al.*, 2011).

Based on a comparative study of two Australian native species, a current hypothesis regarding the regulation of phosphorus resorption is that constitutively expressed phosphate transporters play dual roles in phosphate uptake and resorption (de Campos *et al.*, 2013). AtPht1‐5 was not found to be ideal to test this hypothesis, as the affinity of AtPht1‐5 is unknown and its expression is strictly regulated, with overexpression leading to premature senescence (Nagarajan *et al.*, 2011). In contrast, StPht1‐1, a low‐affinity phosphate transporter from potato, displays near ubiquitous expression in tubers, roots, mature leaves, petioles and flowers and has a suggested Km of 280 μM (Leggewie *et al.*, 1997; Winter *et al.*, 2007). StPht1‐1 was therefore chosen for overexpression in the hybrid poplar P39 to improve plant performance in excess phosphorus conditions as it is a low‐affinity transporter, potentially participating in both uptake and translocation. Elucidating the molecular mechanisms that regulate nutrient translocation and resorption has the potential to impact tree breeding programs for phytoremediation strategies.

## Results

### Atpht 1‐5 insertional mutant

An *Atpht1‐5* Salk line, known to have a T‐DNA insertion located in the third exon (://signal.salk.edu/cgi-bin/tdnaexpress), was first screened for a senescence‐related phenotype. Complementation of the *Atpht1‐5* phenotype with StPht1‐1 would then be used to confirm the role of StPht1‐1 in phosphate translocation. PCR confirmation of the genotypes showed that the Salk lines lacked full‐length gene products present in wild type but amplified shorter length T‐DNA primer products. Although shorter PCR products were indeed present, no transcript was detected from isolated root RNA of homozygous *Atpht1‐5* Salk lines grown on 10 μM H_2_PO_4_^−^.

**Figure 1:**
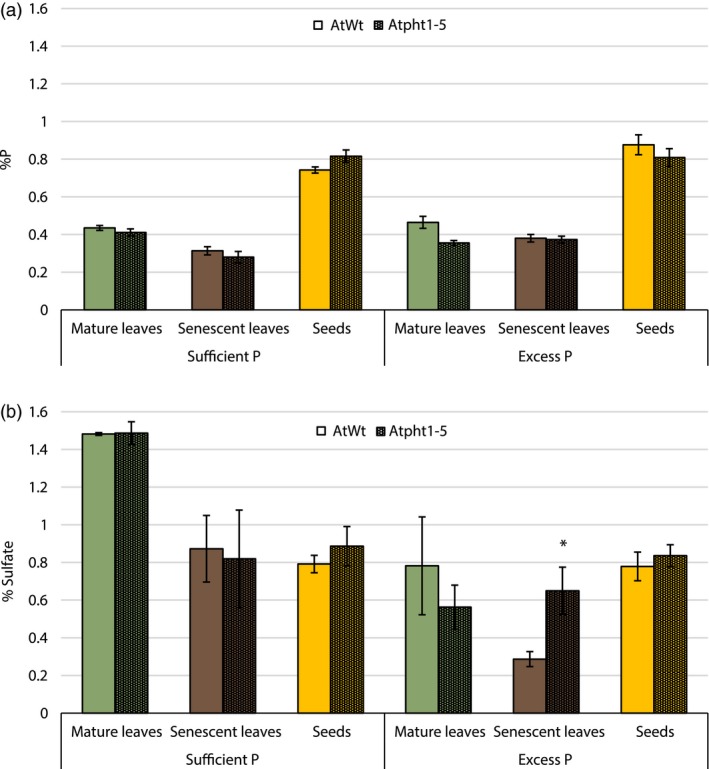
All plants were given 0.1× MS devoid of phosphorus (sufficient P) (*n* = 3) or 0.1× MS with 20 times the usual phosphate concentrations (excess P) (*n* = 6). (a) Phosphorus concentrations (± SEM) as calculated from phosphate and phytic acid in various tissues of wild‐type and *Atpht1‐5* *Arabidopsis thaliana* plants. (b) Sulphate concentrations (± SEM) found in various tissues of wild‐type and *Atpht1‐5* *A. thaliana* plants

In addition, no apparent differences in growth rates or patterns were observed between *Atpht1‐5* and wild‐type plants grown on agar plates, soil or when exposed to optimal and excessive phosphorus conditions. Determination of phosphate concentrations in mature leaves, senescent leaves and seeds also failed to demonstrate observable differences between wild‐type and the T‐DNA line. Overall, mature leaves, senescent leaves, and seeds under phosphorus‐replete conditions contained 0.41%, 0.38% and 0.85% P, respectively (Figure 1a). The majority of phosphorus in seeds was present as phytic acid, while all phosphorus detected in leaf tissue was present as inorganic phosphate. Excess phosphorus treatments did however lead to reduced leaf concentrations of sulphate when compared to plants grown in sufficient phosphorus conditions. Additionally, *Atpht1‐5 *insertional mutants had significantly higher sulphate concentrations in senescent leaves than wild‐type plants (Figure 1b).

### Internal phosphorus concentrations in wild‐type plants

**Figure 2:**
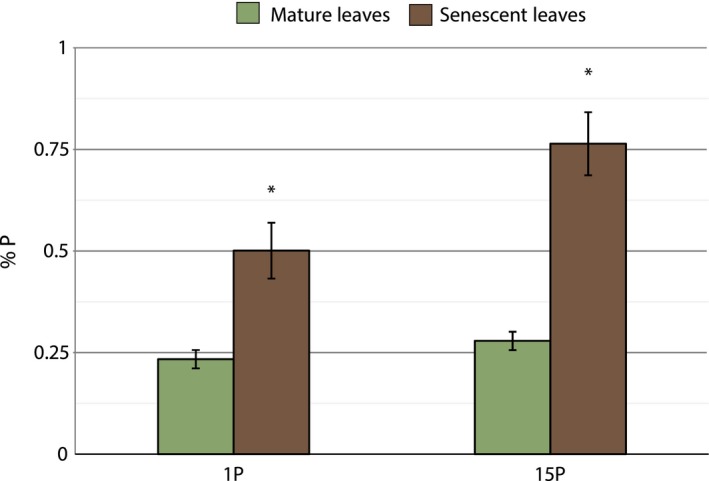
Mean phosphorus concentration (± SEM), as calculated from inorganic phosphate, in hybrid poplar leaf tissue when exposed to two levels of external phosphorus, before (left) and after (right) senescence (*n* = 4). 1P trees were treated with optimal levels of phosphorus, and 15P trees were treated with excess phosphorus 15× above optimal levels. Asterisks (*) indicate *P* < 0.05

Average phosphorus concentrations in mature leaves of wild‐type *A. thaliana* were 0.45% P (Figure 1). In contrast, wild‐type P39 mature leaf concentrations were significantly lower with an average of 0.26% P (Figure 2). Increasing external concentration of phosphate significantly increased final senescent leaf concentrations in both species (Figures 1 and 2).

### StPht1‐1 transcript levels in transgenic lines

**Figure 3:**
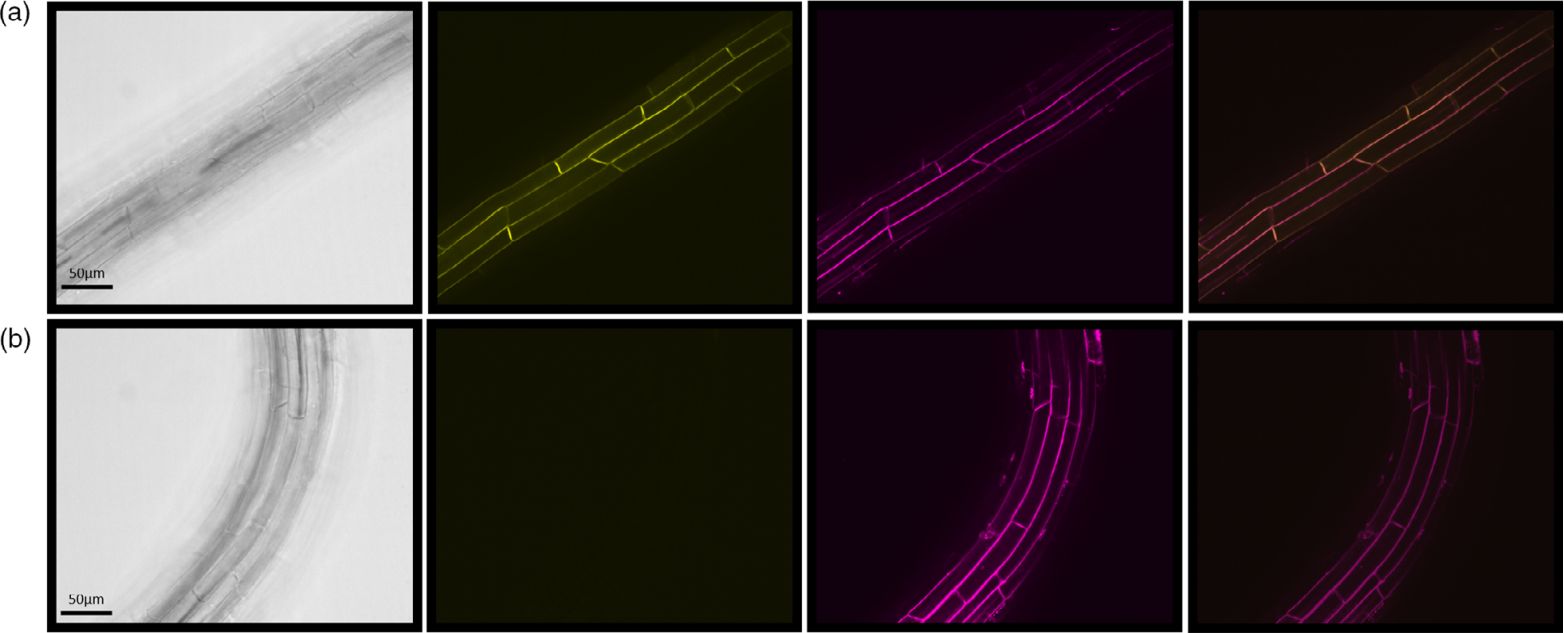
Visualization of yellow fluorescent protein (YFP)‐fluorescent tag on plasma membrane‐bound StPht1‐1 protein in the roots of *Arabidopsis thaliana* transgenic lines. Plasma membrane visualization was achieved using styryl dye FM4‐64. The images are as follows: (a) StPht1‐1 line 3, (b) wild type. From left to right shows the bright‐field image, YFP, FM4‐64 and the merged YFP FM4‐64 image

**Figure 4:**
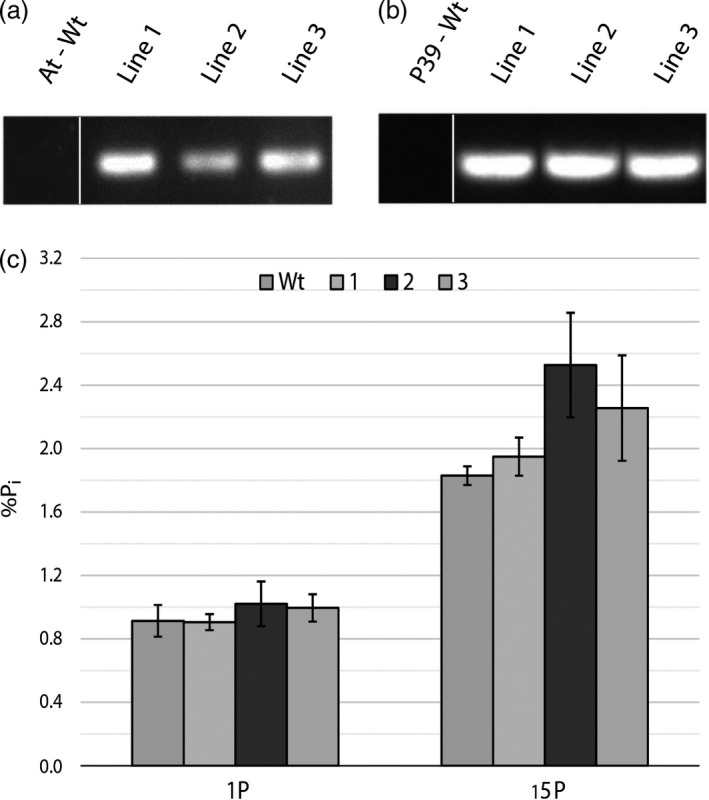
Semi‐quantitative PCR bands showing expression of StPht1‐1 in the root tissue of three *Arabidopsis thaliana* transgenic lines (a), and three P39 transgenic lines (b). Each band represents a pooled sample of three biological replicates. The associated phosphate concentrations found in hybrid poplar root tissue (*n* = 4) at optimal (1P) and excess (15P) levels of external phosphorus (c)

Ten confirmed transgenic *A. thaliana* lines (genomic PCR) were screened to check for the presence and intensity of the yellow fluorescent protein (YFP) fluorescence tag. Visualization of the YFP‐tagged StPht1‐1 protein in the roots of three brightest lines showed the protein localized to the plasma membrane (Figure 3). Transcript abundance in mature leaves relative to ubiquitin was 0.33, 0.75 and 1.38 for selected lines 1, 2 and 3, respectively (Figure S1). In hybrid poplar, StPht1‐1 transcript abundance was analysed in 12 different lines harbouring the 35S::StPht1‐1 insert. From the 12 confirmed lines, the three lines displaying the highest expression were subsequently selected for use in growth trials and were designated as lines 1, 2 and 3, respectively. Relative expression, using ubiquitin as the reference gene, was 0.20, 0.31 and 0.90 for lines 1, 2 and 3, respectively (Figure S1). Moreover, semi‐quantitative PCR performed on root RNA of both *A. thaliana* and P39 using StPht1‐1 primers, produced bright bands in all three lines with no band appearing from the wild‐type controls, as expected (Figure 4a,b).

### Phosphate uptake and resorption in transgenic lines overexpressing StPht1‐1

Under phosphorus‐sufficient conditions, phosphate content between the wild‐type and transgenic *A. thaliana* lines did not differ. Only under excess phosphate conditions did differences appear between the wild‐type plants and line 3 (Table 1). Line 3 had similar phosphate concentration to wild type, but had significantly lower content largely due to lower biomass production. Sulphate content across treatments and phosphate/sulphate resorption efficiencies were unaffected by the insertion of the StPht1‐1 gene.

**Table 1:** Phosphate and sulphate content in transgenic *Arabidopsis thaliana* plants

Line	Mature leaves			Line	Senescent leaves		
	Sufficient P				Sufficient P		
	Phosphate (mg/g)	Phosphate content (mg)	Sulphate content (mg)		Phosphate (mg/g)	Phosphate content (mg)	Sulphate content (mg)
Wild type	12.3	0.39	0.12	Wild type	3.2	0.17	0.01
Line 1	13.4	0.44	0.20	Line 1	2.7	0.16	0.01
Line 2	13.5	0.33	0.13	Line 2	3.9	0.17	0.02
Line 3	12.7	0.35	0.22	Line 3	5.1	0.22	0.01

Line	Mature leaves			Line	Senescent leaves		
	Excess P				Excess P		
	Phosphate (mg/g)	Phosphate content (mg)	Sulphate content (mg)		Phosphate (mg/g)	Phosphate content (mg)	Sulphate content (mg)
Wild type	19.8	1.24	0.23	Wild type	16.9	1.09	0.03
Line 1	21.1	1.03	0.18	Line 1	14.5	0.71	0.01
Line 2	23.2	0.90	0.15	Line 2	18.1	0.77	0.02
Line 3	19.2	0.71*	0.24	Line 3	20.1	0.86	0.01

* A significant difference (*P* < 0.05) when comparing transgenic lines to wild‐type plants (*n* = 6).

**Figure 5:**
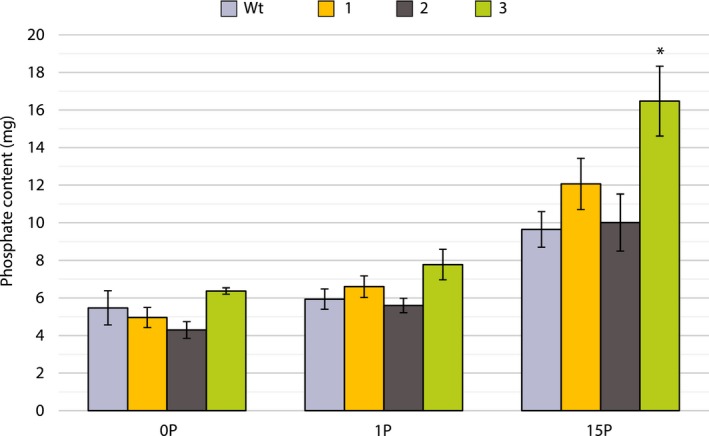
Mean phosphate content (± SEM) of hybrid poplar presenescent leaves when exposed to three phosphate treatments (*n* = 4). 1, 2 and 3 represent the three transgenic lines with highest StPht1‐1 expression. Asterisks (*) indicate *P* < 0.05 when compared against wild‐type trees (Wt)

Although StPht1‐1 transcripts were present in the roots of the transgenic poplar lines, root concentrations of phosphate did not significantly differ from wild type (Figure 4c). Phosphate content in mature leaves of hybrid poplar did not differ when trees were treated with nutrient solutions containing no additional phosphorus (0P) or optimal levels of phosphorus (1P). However, when exposed to 15× optimal level of phosphorus (15P), wild type, line 1 and line 2 accumulated 1.7× more phosphate in a mature leaf, while line 3 had a 2.1‐fold increase in phosphate content and accumulated a significantly higher amount of phosphate than wild‐type trees and the other two lower‐expressing transgenic lines (Figure 5).

**Figure 6:**
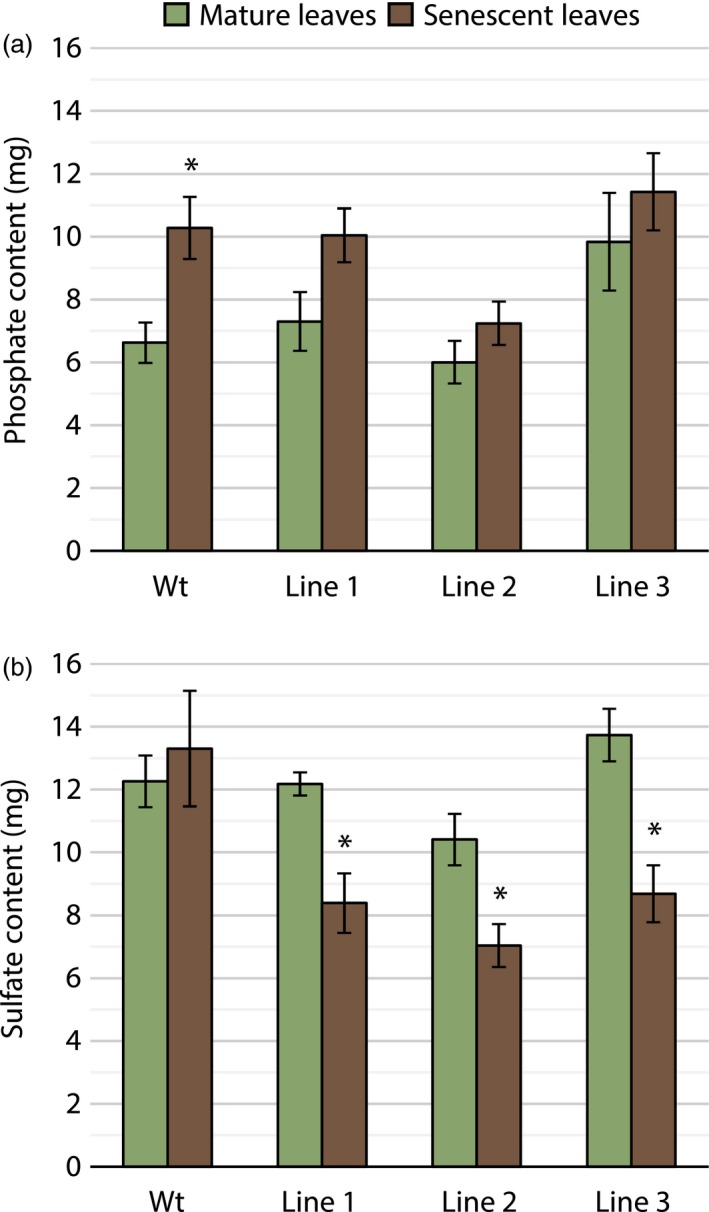
A comparison of phosphate and sulphate content in wild‐type hybrid poplar and each of the three transgenic poplar lines. (a) Mean phosphate content (± SEM) of mature leaves and senescent leaves at the control treatment (100N:13P; *n* = 4). (b) Mean sulphate content (± SEM) of mature leaves and senescent leaves. Phosphate treatments did not affect sulphate content resulting in the pooling of biological replicates across treatments (*n* = 10). Asterisks (*) indicate *P* < 0.05

For an assessment of resorption efficiencies, phosphate content in mature and senescent leaves was compared. Wild‐type trees showed significantly higher content in senescent leaves, while all transgenic lines had similar values for mature and senescent leaf content (Figure 6a). Interestingly, transgenic lines 1, 2 and 3 had 31.3%, 32.3% and 36.8% sulphate resorption efficiencies respectively, while wild‐type trees did not show significant resorption of sulphate (Figure 6b). An assessment of mature leaves showed an average chlorophyll concentration index (CCI) of 43.1, while all senescent leaves had a CCI < 5.0.

## Discussion

The lack of a phosphorus related phenotype in *Atpht1‐5* lines was unexpected, as a previous study showed that Atpht1‐5 seedlings have a higher shoot phosphorus content under phosphate‐replete conditions (Nagarajan *et al.*, 2011). Overexpression of AtPht1‐5 also resulted in elevated phosphate accumulation in siliques and induced premature senescence, implying that AtPht1‐5 plays a role in phosphate remobilization from source to sink tissues (Nagarajan *et al.*, 2011). This effect on remobilization is refuted by the data shown in Figure 1 in this study, where an* Atpht1‐5* knockout mutant, with confirmed lack of transcript, did not result in a higher senescent leaf phosphorus concentration or reduced phosphorus accumulation in the seeds. This is assumed to be due to the redundant function of phosphate transporters in *Arabidopsis,* which has already been shown to occur extensively in roots (Ayadi *et al.*, 2015). The full extent of redundancy in transporters involved in phosphate remobilization and resorption in *Arabidopsis* is unknown and may involve other phosphate transporters such as PHO1 (Stigter and Plaxton, 2015). The increased sulphate concentrations during senescence in *Atpht1‐5* requires further investigation to elucidate whether sulphur storage is being affected across the many organic sulphur fractions within the plant and how the inhibition of phosphate transport affects sulphate remobilization.

*Arabidopsis thaliana* ecotype ‘Columbia’ and the poplar hybrid P39 are suitable candidates in which to express StPht1‐1 for a variety of reasons. Under sufficient external phosphorus conditions, both species had internal phosphate levels of ≤0.4% P, which is consistent with values common to crop plants grown under optimal conditions (Marschner, 2012). When exposed to weekly treatments of excess phosphate, *A. thaliana* showed no increase in cellular phosphate concentrations, while the hybrid poplar trees showed minimal increases in phosphate concentration, implying that they have little propensity for accumulating excess phosphate. Poor resorption proficiencies were observed in both species, well above the threshold of 0.05% P (Killingbeck, 1996) (Figures 1 and 2). As such, these inherent biological responses make both species suitable for constitutive overexpression of an exogenous low‐affinity phosphate transporter, as their low resorption and observed status as low accumulators allowed for gains in both parameters to be assessed.

Nutrient remobilization and senescence are both complex events that are initiated by many environmental and metabolic factors. As a consequence, very little is known about the mechanisms regulating the efflux of anions, such as sulphate and phosphate, from the cell (Himelblau and Amasino, 2001; Smith *et al.*, 2000). An inherent difficulty in the study of senescence is that genes involved in nutrient transport likely play a role in plant growth and development. This means that knockout mutants could exhibit drastic growth phenotypes, masking the effects on senescence. Proof of function in senescence would therefore require mutants to be complemented until the initiation of senescence. Redundancy may also be an issue, with the potential for multiple gene knockouts being required to produce a discernible phenotype (Himelblau and Amasino, 2001). This is not currently feasible in high‐utility species such as poplar, due to their perenniality, but may be realized in the near future with the advent of CRISPR/Cas9 as a genome‐editing tool in plants, including poplar (Zhou *et al.*, 2015). To circumvent these difficulties, we chose to address a hypothesis put forward by de Campos *et al. *(2013), who suggested that constitutively expressed transporters could play dual roles in uptake and resorption. A well‐characterized low‐affinity transporter was therefore chosen to avoid the potentially toxic accumulation of phosphorus brought on by the overexpression of high‐affinity transporters in roots (Smith *et al.*, 2000).

Constitutive, plant‐wide expression of StPht1‐1 had different effects in the two plant species examined in this study. In the annual *A. thaliana*, plants expressing the exogeneous StPht1‐1 and grown under high‐phosphorus treatments had decreasing phosphate content, with the most significant decrease occurring in the line displaying the highest transcript abundance (Table 1). This is caused by the apparent reductions in biomass rather than concentration, suggesting accumulation to the point of toxicity (Teng and Timmer, 1990). As for resorption, only treatment affected the phosphate resorption efficiency, suggesting that sink strength is a driver of phosphorus resorption in *A. thaliana* (Table 1). Overall, expression of StPht1‐1 did not increase the performance of *A. thaliana* in excess phosphate conditions.

In the perennial tree species, poplar, transgenic lines expressing StPht1‐1 displayed an increase in leaf phosphate content when the trees were exposed to high levels of external phosphate (Figure 5). This suggests that constitutive expression of an exogenous low‐affinity Pht1 transporter can reduce the ability of hybrid poplar to moderate phosphate uptake when external phosphate concentrations are high, making it a positive modification for the use of hybrid poplar in phytoremediation applications. No measurable differences in biomass were observed between lines, indicating that no toxicity symptoms were induced by the increased accumulation of phosphate (Teng and Timmer, 1990). A positive effect on resorption was also observed in all three transgenic lines compared with wild‐type trees, both in terms of phosphate and sulphate. Minor gains were achieved in phosphate resorption in the control treatment, where all three lines showed similar phosphate before and after senescence, while wild‐type trees had a significantly higher phosphate content in senescent leaves (Figure 6a). Unexpectedly, sulphate resorption efficiencies in the transgenic lines increased from 0% in the control to 31.1%, 32.4% and 36.8% in lines 1, 2 and 3, respectively (Figure 6b). This indicates that barriers to resorption in hybrid poplar may be, in part, the controlled efflux of nutrients from the vacuole through anion channels. Efflux from the vacuole is passive and occurs along the individual anion electrochemical gradients. For phosphate in particular, Pratt *et al. *(2009) demonstrated that when methylphosphonate (MeP), a nonmetabolically active phosphate mimic, was supplied to phosphate‐deficient cells, it prevented the efflux of phosphate from the vacuole. During senescence, the degradation of the organic phosphorus pool may maintain high cytosolic phosphate, thus inhibiting the release of vacuolar stores. Increasing the rate of phosphate efflux from the cytosol through the expression of StPht1‐1 may have allowed for greater efflux of anions from the vacuole. However, why sulphate would be the preferred anion for resorption with the up‐regulation of a phosphate transporter is currently unknown.

The mobility and transport of anions across the tonoplast has historically not been well understood. Recent studies in rice and *Arabidopsis* have begun to characterize the elusive anion channels, including AtPht5‐1 and OsSPX‐MFS1 that mediate the influx of anions (with phosphate as the preferred anion) across the tonoplast. Overexpression of these channels has led to increased sequestration within plant vacuoles (Liu *et al.*, 2015, 2016). Like the Pht1 family of transporters, they are a part of the major facilitator superfamily (MFS) which is one of the largest families of membrane transporters (Pao *et al.*, 1998). However, unlike Pht1 proteins, they contain a well‐conserved SPX domain, a sequence which has been linked to the identification of key proteins in phosphate homeostasis in all major eukaryotes, ranging from *Caenorhabiditis elegans*, to *Drosophila melanogaster*, to *A. thaliana and* to *Homo sapiens* (Secco *et al.*, 2012). In plants, studies in *Arabidopsis* and rice have been at the forefront of this research, with a vast array of effects documented from the mis‐regulation of SPX‐MFS genes. Localization of these proteins in the cell is also quite variable (Secco *et al.*, 2012). To date, the most promising of the SPX‐MFS proteins for improving the plant phosphate resorption is OsSPX‐MFS3, a constitutively expressed tonoplast‐bound, low‐affinity phosphate transporter that can facilitate phosphate efflux from the vacuole based on external pH and phosphate concentrations. For example, overexpression of OsSPX‐MFS3 in rice resulted in significantly lower vacuolar phosphate concentrations in both leaves and roots (Wang *et al.*, 2015). A corresponding gene in *Arabidopsis* has yet to be discovered, and phylogenetic analysis of SPX‐MFS homologues between the two species suggests that monocot and eudicot genes may have evolved independently, possibly resulting in functional variation in this class of proteins. The phylogenetic analysis of SPX‐MFS DNA coding sequences in multiple plant species also resulted in *P. trichocarpa* SPX‐MFS grouping with those from tomato (*Solanum lycopersicum* L.) and grapevines (*Vitis vinifera* L.), separate from those found in *Arabidopsis*, suggesting different selective pressures acting on phosphate homeostasis in the two species used in this study (Liu *et al.*, 2016).

## Summary

Constitutive plant‐wide expression of the low‐affinity MFS transporter StPht1‐1 demonstrated that the barriers and controls of resorption differ in *A. thaliana* and hybrid poplar. In *A. thaliana*, the overexpression of a low‐affinity root phosphate transporter caused toxicity symptoms in plants grown in high‐phosphorus conditions. No changes to resorption were observed between wild‐type and the transgenic lines, suggesting that nutrient status and sink strength have a greater effect on nutrient resorption than the expression of StPht1‐1. In hybrid poplar, plant‐wide abundant expression of StPht1‐1 increased leaf phosphate content in excess phosphorus conditions. Improved phosphate resorption efficiency, and to a larger extent, sulphate resorption efficiency were apparent in all transgenic lines during senescence. Given that the effects of age, sink strength and timing of abscission on phosphorus resorption in tree species are negligible (Chapin and Moilanen, 1991; Killingbeck *et al.*, 1990), these results suggest that in phosphorus‐replete conditions, resorption is limited, in part, by the rate of export of phosphate from the cell. Of greater significance may be the efflux of phosphate reserves out of the vacuole. For the purposes of increasing phosphate remobilization and improving the phosphorus phytoremediation potential in poplar, increased content under excess conditions with no effect on biomass production is a positive step forward. However, further gains to phosphate resorption efficiencies and proficiencies can likely be achieved with additional, refined strategies for altering leaf phosphate status.

## Materials and methods

### Confirmation of T‐DNA insertional mutant

Seeds of a putative T‐DNA insertional line affecting AT2G32830 (AtPht1‐5) were obtained from the Arabidopsis Biological Resource Center (Alonso *et al.*, 2003). The line employed in this study has been annotated as SALK_106359.51.45.X. Seeds were planted directly onto soil and grown at a light intensity of 175 μmol/m^2^/s for 16‐h days at 22 °C and 8‐h night cycles at 20 °C. Once plants had approximately five unfurled true leaves, genomic DNA was extracted from a single leaf for PCR confirmation of the presence of the T‐DNA insertion. Primers used for genotyping can be found in Table S1. After the insertional mutant was confirmed homozygous, lines were screened for discernible growth phenotypes, according to Bolle (2009).

### Cloning of StPht1‐1

Due to the absence of introns in the gene encoding StPht1‐1 (PGSC0003DMG400017226), the low‐affinity potato phosphate transporter was cloned directly from genomic DNA. Potato DNA was isolated using a CTAB extraction method (Ghislain *et al.*, 1999) from the leaf tissue of *Solanum tuberosum* cv. Russet. A blunt‐end PCR product of the StPht1‐1 gene was produced using a forward primer with the required 5′ CACC overhang for Directional TOPO^®^ cloning into the pENTR Gateway^®^ entry vector. The resulting pENTR–StPht1‐1 plasmid was transformed into chemically competent cells of the DH5α strain of *Escherichia coli* (Thermo Fisher Scientific, Carlsbad, CA). After selection of positive colonies and isolation of the pENTR–StPht1‐1 plasmids, three LR recombination reactions were completed to insert the cloned StPht1‐1 into multiple destination vectors (Thermo Fisher Scientific). Two destination vectors were for use in *A. thaliana* (pH7WGY2 and pH7YWG2), and one destination vector was for use in the P39 poplar hybrid (pK2GW7). The pH7WGY2 plasmid contains an N‐terminal YFP tag, while the pH7YWG2 has a C‐terminal YFP tag. pK2GW7 does not contain a sequence for a fluorescent protein tag. An alternate reverse primer was used to remove the StPht1‐1 stop codon prior to cloning into the pH7YWG2 destination vector so the transcription of the YFP tag would not be impeded. In two separate transformation events, the destination vectors pH7WGY2 and pH7YWG2 were transformed into DH5α and subsequently into *Agrobacterium tumefaciens* strain GV3101*.* pK2GW7 was transformed into the *A. tumefaciens* strain EHA101.

### Plant transformations

*Arabidopsis thaliana* wild‐type plants were transformed using the floral dip method as described in Zhang *et al. *(2006). Insertion of StPht1‐1 was confirmed using PCR of genomic DNA using primers listed in Table S1 and was grown for successive generations until homozygous lines were attained. Poplar hybrid P39 was clonally propagated in woody plant medium (WPM), and leaf disks were transformed based on a protocol adapted from Coleman *et al. *(2006). The resulting plants that grew successfully on kanamycin selection media were then subject to genomic DNA screening by PCR for confirmation of the StPht1‐1 insertion, using primers listed in Table S1.

### Transgenic line selection

From the 12 successful poplar transformants and six homozygous *A. thaliana* StPht1‐1 lines with visible YFP fluorescence, expression levels of the exogenous potato phosphate transporter were quantified using qPCR using the primers listed in Table S1. First, RNA was extracted from leaf tissue using TRIzol™ reagent and manufacturer’s protocol (Thermo Fisher Scientific), followed by a DNase clean‐up (TURBO™ DNA‐free™ Kit; Thermo Fisher Scientific) and cDNA synthesis (iScript™ Select cDNA Synthesis Kit; Bio‐Rad, Hercules, CA). The resulting cDNA was diluted to 1/10 of the original concentration. The final 10 μL qPCR included SsoFastTM EvaGreen® Supermix (Bio‐Rad), reference or target gene qPCR forward and reverse primers and 1 μL of diluted cDNA. Serial dilutions were performed to calculate the primer efficiencies of the reference genes (UBQ and TIF) and the target gene (StPht1‐1). UBQ was chosen as the reference gene, as the primer efficiency was closest to that of StPht1‐1. qPCRs were completed on the Bio‐Rad CFX96TM real‐time PCR system following the Bio‐Rad optimized cycling conditions stated for cDNA with an annealing/extension step at 55 °C for 5 s (Bio‐Rad, 2016). Three *A. thaliana* lines were subsequently used in the growth chamber experiments, while three poplar lines with the highest StPht1‐1 transcript abundance were bulked on WPM, until numbers were sufficient for greenhouse experiments.

### Growth trials

For all *A. thaliana* growth trials, seeds were surface‐sterilized with 70% ethanol and 10% bleach and then left to stratify in the dark at 4 °C for two days. Thereafter, the seeds were planted directly onto soil with four plants per pot in a randomized block design within a tray. One tray was subjected to a 0.1× MS with no phosphorus (sufficient P), while a second tray was given 0.1× MS with 20× regular phosphorus amounts (excess P). All watering was done using the treatment solution. Plants were grown at a light intensity of 175 μmol/m^2^/s with 16‐h days at 22 °C and 8‐h nights at 20 °C. On the fourth week, before plants began to bolt, two plants from every pot were harvested and lyophilized. Dry weights of the plant were taken before the plant was ground using a FastPrep machine for 20–40 s at speed setting 4.0. Ten milligrams of dried tissue was then weighed and acid‐extracted according the protocol outlined in Bentsink *et al. *(2002), for phosphate and sulphate determination using high‐performance liquid chromatography (HPLC) fit with an IonPac AS‐11 column (4 × 250 mm) (Thermo Fisher Scientific) and a conductivity detector. The remaining plants were then watered, as needed, until all were visibly brown, dried and had set seed, at which time they were considered to have fully senesced. Once senesced, rosettes were collected and lyophilized before dry weight was taken and the same acid extraction performed.

Hybrid poplar were grown in the University of British Columbia Horticulture Greenhouse (Vancouver, Canada) from November 2016 to March 2017. The three lines with the highest transcript abundance were grown alongside wild‐type trees in a randomized block design and were given one of the following nutrient ratios: 100N:0P, 100N:13P, 100N:195P mg/L. These nutrient loads correspond to the 0P, 1P and 15P treatments, respectively. After ten weeks of growth, the ninth fully mature leaf from each tree was flash‐frozen in liquid nitrogen, lyophilized and ground using the 2010 GenoGrinder^®^ tissue homogenizer (SPEX^®^ SamplePrep, Metuchen, NJ) at 1600 rpm for 30 s. At this time, trees were then moved outside to senesce under normal growing conditions. Once leaves were visually >80% yellow, a CCI measurement was taken on the top right‐hand side of the leaf using a Opti‐Sciences CCM‐200 plus chlorophyll meter prior to the specific leaf being flash‐frozen in liquid nitrogen. Leaves were only collected if they abscised when touched. Ground tissue was acid‐extracted based on a protocol adapted from Bentsink *et al. *(2002) and determination of phosphate and sulphate completed using HPLC coupled with a conductivity detector. Samples from leaves and roots were also taken to confirm StPht1‐1 expression in both tissues.

### Statistical analysis

R version 3.3.2 (R Core Team, 2016) was used to fit linear mixed‐effects models with the packages lmerTest (Kuznetsova *et al*., 2015) and nlme (Pinheiro *et al.*, 2016). ANOVA was used to compare statistical models and to test the significance of the categorical predictors and their interactions. Multiple comparisons were completed using the Tukey method as a *post hoc* test to identify significance between sample means.

## Conflict of interest

Authors declare no conflict of interest.

## Author contributions

L.M.D.R. and S.D.M. conceived and designed the project; L.M.D.R. performed the experiments and analysed the data; L.M.D.R. and S.D.M. wrote and edited the manuscript, respectively.

## Supporting information


**Figure S1** Relative expression (± SEM) of StPht1‐1 using ubiquitin as the reference gene in both *Arabidopsis thaliana* and hybrid poplar. (A) Expression of StPht1‐1 in three highest expressing transgenic lines of *A. thaliana* (*n* = 3). (B) Expression of StPht1‐1 in the three highest expressing transgenic lines of the poplar hybrid (*n* = 3).
**Table S1** Primer sequences and their respective uses.
