# Supplementary material for: Biotechnological mechanism for improving plant remobilization of phosphorus during leaf senescence
Source: Plant Biotechnol J. 2019 Aug 27;18(2):470–8. doi: 10.1111/pbi.13212 (PMC6953190; doi:10.1111/pbi.13212)
Supplement: Supplementary file 1 — Figure S1 Relative expression (± SEM) of StPht1‐1 using ubiquitin as the reference gene in both Arabidopsis thaliana and hybrid poplar. (A) Expression of StPht1‐1 in three highest expressing transgenic lines of A. thaliana (n = 3). (B) Expression of StPht1‐1 in the three highest expressing transgenic lines of the poplar hybrid (n = 3). Table S1 Primer sequences and their respective uses. [file PBI-18-470-s001.docx]

**Figure S1:** Relative expression (± SEM) of StPht1-1 using ubiquitin as the reference gene in both *A. thaliana* and hybrid poplar. A) Expression of StPht1-1 in three highest expressing transgenic lines of *A. thaliana* (n = 3). B) Expression of StPht1-1 in the three highest expressing transgenic lines of the poplar hybrid (n = 3).


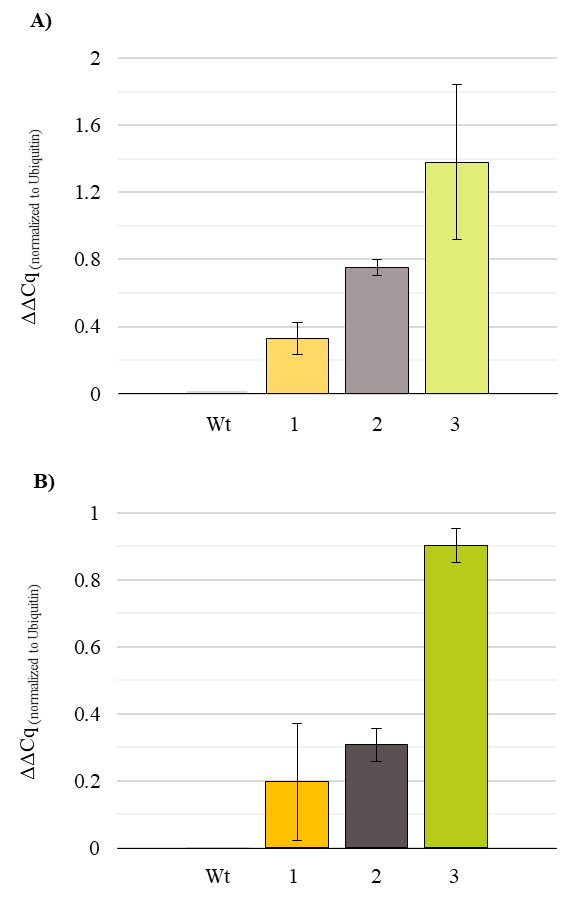


**Table S1:** Primer sequences and their respective uses.

| **Use** | **Primer name** | **Sequence** |
| --- | --- | --- |
| Genotyping (TDNA mutant) | SALK_106359C LP | TCGGGTCTGGTCTATCATTTG |
|  | SALK_106359C RP | GACAAGTCGATCTCAACCTCG |
|  | SALK_LB Primer | ATTTTGCCGATTTCGGAAC |
| Cloning | StPht1-1f | CACCATGGCGAACGATTTG |
|  | StPht1-1r (Stop Codon) | TTAAACAGGAACTGTCCTTCCAC |
|  | StPht1-1r (No Stop Codon) | AACAGGAACTGTCCTTCCAC |
| Genotyping (transgenics) | CStPht1-1f | GATGCTCTTGCTTCAACAG |
|  | T35Sr | CCCTTATCTGGGAACTACTCAC |
| qPCR | CStPht1-1f | GATGCTCTTGCTTCAACAG |
|  | CStPht1-1r | CTTGCAACACCTTGGACATG |
|  | PtUBQf | GTTGATTTTTGCTGGGAAGC |
|  | PtUBQr | GATCTTGGCCTTCACGTTGT |
|  | AtUBQ_5_f | CAGCTCCACAGGTTGCGTTA |
|  | AtUBQ_5_r | CAAGCCGAAGAAGATCAAGCACAAG |
